# Dietary restriction as a potential neuroprotective intervention: a narrative review of its impact on neuroinflammation across neurodegenerative diseases and other neurological disorders

**DOI:** 10.3389/fnut.2026.1731416

**Published:** 2026-04-13

**Authors:** Buhui Liu, Yating Zhang, Wenxin Deng, Ya-nan Zhou, Yuhan Cao, Lie Xiong, Ruoyi Shen, Simon Ming-Yuen Lee, Jinsong Bian, Yaqi Bian

**Affiliations:** 1 Jiangsu Key Laboratory of Brain Disease and Bioinformation, Research Center for Biochemistry and Molecular Biology, Xuzhou Medical University, Xuzhou, China; 2 National Experimental Demonstration Center for Basic Medicine Education Department of Clinical Medicine, Xuzhou Medical University, Xuzhou, China; 3 The Second Clinical Medical School, Xuzhou Medical University, Xuzhou, China; 4 School of Life Sciences, Xuzhou Medical University, Xuzhou, China; 5 The First Clinical Medical School, Xuzhou Medical University, Xuzhou, China; 6 Department of Food Science and Nutrition, The Hong Kong Polytechnic University, Hung Hom, Hong Kong SAR, China; 7 Department of Pharmacology, School of Medicine, Southern University of Science and Technology, Shenzhen, Guangdong, China

**Keywords:** dietary restriction (DR), gut-brain axis, neurodegenerative disease, neuroinflammation, neurological disease, neuroprotection

## Abstract

Dietary restriction (DR) involving chronic or intermittent calorie/nutrient reduction without malnutrition, delays neurological disease progression. Decades of research across *in vitro* models, animal studies, and clinical trials provide preclinical evidence for a potential role of DR in modulating multiple mechanisms underlying CNS disorders. Interactions between caloric intake, meal frequency, diet composition, and the gut microbiome regulate specific metabolic pathways governing cellular, tissue, and organ homeostasis as well as inflammatory processes during neurodegenerative and neurological diseases. In this review, we synthesize evidence on the role of DR in modulating neuroinflammation and related mechanisms within a selected set of neurodegenerative and neurological disorders, aims to provide a consolidated evidence base and perspective on the potential of DR as an adjunctive strategy for the future therapeutic investigations.

## 1 Introduction

Dietary restriction (DR) is defined as the reduction of food intake chronically or intermittently without malnutrition. Preclinical studies indicate that DR can delay the onset and progression of age-related pathologies, including those affecting the nervous system (1, 2). Common approaches for DR include an overall reduction in calories or the restriction of specific nutrients. There are many forms of DR protocol, which include, but are not limited to, intermittent fasting, diets including a defined overall number of calories, and the restriction of specific nutrients. The putative health-promoting effects of DR in neurological contexts are considered to arise from its broad influence on fundamental processes such as metabolism, oxidative stress, and inflammation (2, 3). Experimental studies have demonstrated that inflammatory pathways can be regulated by dietary manipulations to prevent the accumulation of molecular and cellular damage in neurological diseases (4). Increasing evidence of the interactions between the activation of inflammatory pathways and energy metabolism in the central nervous system (CNS) indicates a complex relationship between these two physiological processes. In addition, studies have proven that glucose deprivation can increase central insulin resistance and influence mitochondrial function in the brain, these are key factors in the development of neurodegenerative diseases and neuropsychiatric disorders. Thus, scientists hypothesized that neuroprotective effects could be achieved, at least in part, by the reconstruction of metabolism and energy balance through DR (5–7). This has further led to the hypothesis that DR might confer neuroprotection, in part, by recalibrating metabolism and energy homeostasis, thereby exerting antineuroinflammatory effects.

Neuroinflammation refers to the coordinated response of the innate immune system in the brain to detrimental stimuli and injuries, including trauma, infections, the accumulation of abnormal proteins, and damage caused by genetic mutations, and aims to protect and defend the CNS (8). Microglia and astrocytes are the two primary glial cells in the CNS that are responsive to harmful stimuli (9). Microglia and astrocytes are the two primary glial cells in the CNS that are responsive to harmful stimuli (10, 11). Neuroinflammation usually correlates with the breakdown of the immune system in the CNS, along with the increasing penetration and permeability of the blood–brain barrier (BBB). Under these conditions, the vulnerable BBB allows soluble proinflammatory factors to enter the CNS; this process is accompanied by immune cell trafficking (12). These proinflammatory factors could directly interact with neurons and glial cells to initiate the inflammatory process to remove the threat. At this point, downstream anti-inflammatory pathways are activated to restore the functionality of damaged regions and also repair tissue integrity. Generally speaking, the recovery of tissue integrity and its corresponding functionality could be accomplished within a certain timeframe. This process is referred to as acute neuroinflammation, an event that is commonly associated with infection and trauma in the CNS (13, 14). However, inflammatory factors can be persistent, particularly those associated with certain neurodegenerative diseases and neuropsychiatric disorders (15). Consequently, the processes responsible for anti-inflammation and the restoration of brain function can slow down, resulting in chronic neuroinflammation (16). The precise role of neuroinflammation, as a primary driver or a secondary consequence, in neurological diseases remains an active area of research investigation (17). Accumulating evidence has proven that chronic neuroinflammation could represent one of the most crucial pathogenic mechanisms in the progression of multiple neurological diseases, including, but not limited to, Alzheimer’s disease (AD), Parkinson’s disease (PD), multiple sclerosis (MS), amyotrophic lateral sclerosis (ALS), depression, anxiety, and epilepsy (18–21). Therefore, neuroinflammation has emerged as a pivotal player in the progression of neurological diseases (22). In this narrative review, we aimed to synthesize and critically evaluate existing evidence on the effects of DR on neuroinflammation within a selected group of neurodegenerative and neurological disorders. By examining findings from preclinical models (which constitute the majority of the mechanistic evidence) alongside the more limited and heterogeneous data from human clinical studies, our review provides a balanced perspective on the potential of DR as a modulatory intervention, highlighting its promising mechanisms and the remaining substantial gaps in clinical translation.

**Figure 1:**
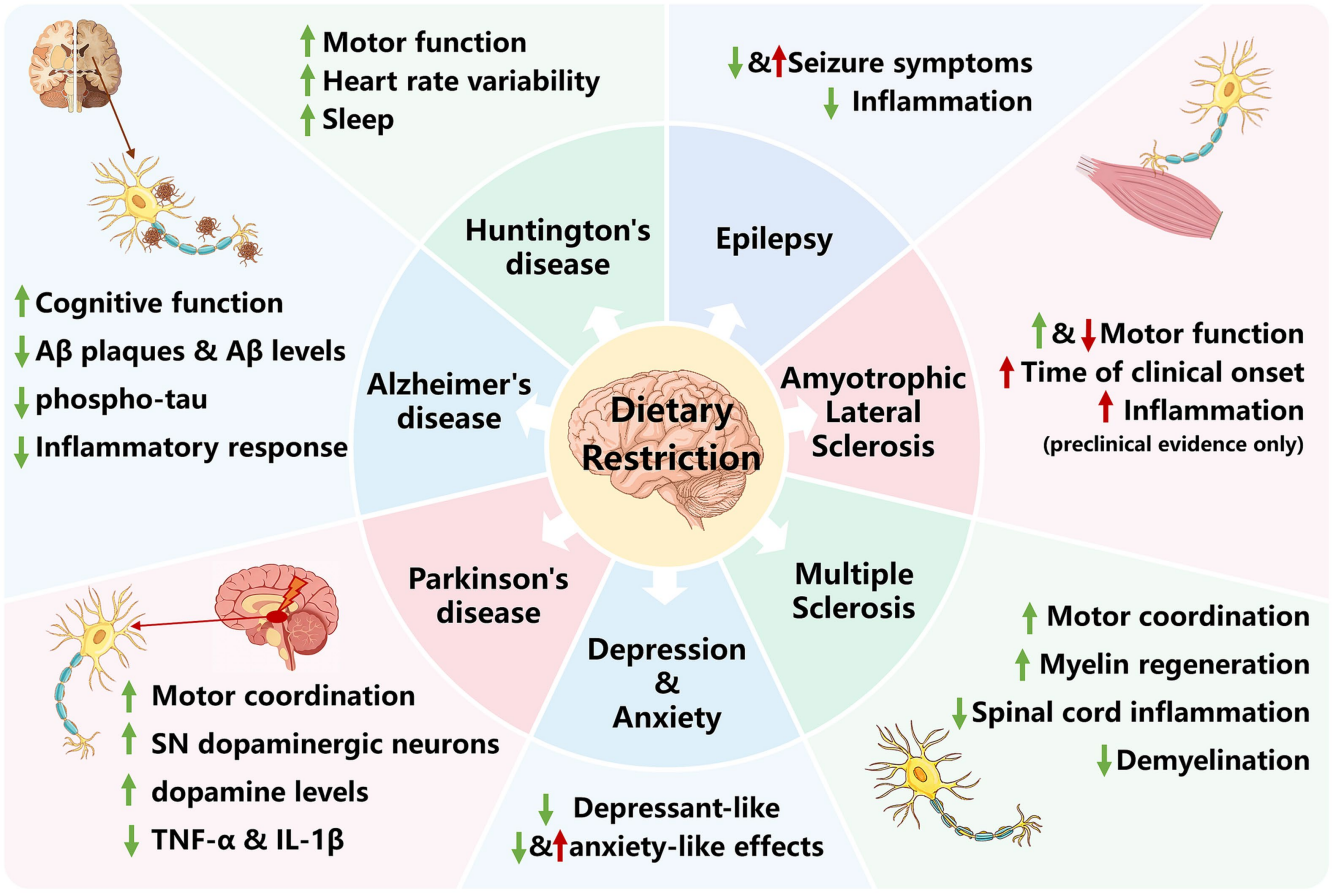
The context-dependent role of DR in neuroinflammation. Graphical summary of the evidence that the effects of DR on neuroinflammation are not generalizable but are heterogeneous across neurodegenerative and neurological diseases. Different disease environments channel DR-induced mechanisms toward divergent outcomes, explaining the lack of unified beneficial effects and indicating the critical importance of disease-specific investigation. Color-coded arrows (green for protective or beneficial modulation, and red for detrimental or harmful modulation) illustrate the divergent effects.

In this review, we synthesize evidence from major scientific databases, including Web of Science, Scopus, PubMed, Google Scholar, and ScienceDirect over the last 25 years (from 2000 to 2024), to provide a comprehensive and critical appraisal of the role of DR in modulating neuroinflammation across most neurodegenerative diseases and selected neurological disorders (Figure 1). We focused on elucidating key mechanistic pathways and biological markers, evaluating translational evidence from preclinical models to clinical trials, and examining the heterogeneous and disease-specific outcomes of DR. The literature search employed a combination of key terms related to intervention methods (e.g., “dietary restriction,” “calorie restriction,” “intermittent fasting”) and outcomes (e.g., “neuroinflammation,” “neurodegeneration”) across specific diseases (e.g., “Alzheimer’s disease,” “Parkinson’s disease,” “amyotrophic lateral sclerosis,” “epilepsy,” “depression”). Retrieved articles were screened and organized in a two-step process. First, titles and abstracts were reviewed for relevance. Subsequently, the full texts of potentially eligible articles were assessed against predefined inclusion criteria (original research articles focusing on DR and neuroinflammation in the selected neurological diseases, including preclinical and clinical studies) and exclusion criteria (reviews without original data, studies not focusing on the CNS, and articles for which the full text was unavailable or was not written in English). Eligible studies were categorized thematically by disease and mechanistic focus to structure this narrative review.

## 2 Neuroinflammation and neurodegenerative diseases

### 2.1 Neuroinflammation in the aging brain

As one of the most harmful effects of aging in the CNS, functional brain deterioration cannot be neglected. Low levels of chronic systemic inflammation in the CNS are believed to be associated with the aging process. Evidence has shown that the age-related functional decline of the brain is closely correlated with neuroinflammation and active glial cells in the CNS. Gliosis, a neuroinflammatory hallmark related to the proliferation and activation of glial cells (microglia, astrocytes, and oligodendrocytes), occurs in response to a number of insults in the brain. Under this condition, microglia, which act as the dominating innate immune cells in the CNS, express high levels of MHCII; in addition, astrocytes express increased levels of glial fibrillary acidic protein (GFAP) upon aging (23, 24). Henry et al. reported evidence that microglia from the brains of aged mice were more sensitive to inflammatory stimuli than those from younger mice (25). Consistent with this, increased levels of inflammatory cytokines, including interleukin-1 beta (IL-1β), tumor necrosis factor alpha (TNF-*α*), and interleukin-6 (IL-6) have been detected in aged brains (26–28). Furthermore, as a defense mechanism against inflammation, reduced levels of certain immune regulatory molecules have been detected in the brains of aged rodents, including interleukin-10 (IL-10) and interleukin-4 (IL-4) (29). Multiple brain regions, including the hippocampus and cortex, are known to be involved in the inflammatory response. As the hippocampus is responsible for memory and cognition, pathological changes (the aggregation of abnormal proteins and the hyperphosphorylation of functional proteins) followed by a chronic inflammatory response have been detected in this part of the brain in neurodegenerative diseases (30, 31). In this respect, neuroinflammation has been proven to play a pivotal role in AD. Research has indicated that neuroinflammation might represent the direct pathogenic factor responsible for AD, or that this condition could be indirectly induced by the accumulation of Aβ and hyperphosphorylated tau protein (32, 33). The aggregation of *α*-synuclein also represents a pathological hypothesis for PD in that neuroinflammation has been found to be correlated with the aggregation of α-synuclein in PD (34, 35).

Previous studies have indicated that dietary interventions could be of benefit to the degenerative changes in the CNS induced by aging. For instance, in the brains of senile mice, evidence showed that inflammatory signaling was closely associated with aging and that a long-term ketogenic diet could activate several antiaging genes (36). Jantsch et al. (37) also reported that a 30% calorie restriction (CR) for 5 weeks reduced neuroinflammation and ameliorated memory impairment in obese aged rats. In another study involving 19–31-year-old rhesus monkeys, 30% CR for 12–17 years reduced the circulating levels of IL-6, a factor that is known to be associated with brain atrophy (38). Other studies have focused on the effects of different DR methods on ameliorating neuroinflammation and cognitive deficits in more specific neurodegenerative diseases such as AD and PD. As outlined herein, DR demonstrates significant benefits for the mitigation of age-related functional decline in the brain and for attenuating associated neuroinflammatory responses in neurodegenerative disorders.

### 2.2 Different approaches of DR

Although the approaches utilized for DR are highly diverse, the central process involves reducing the amount of food intake or altering the frequency or timing of food intake, without causing any malnutrition. There are a number of DR approaches, including, but not limited to, a 20–50% reduction of daily food intake or calorie intake (in animals fed *ad libitum*) without a change in meal frequency, alternate day fasting and refeeding, or time-restricted feeding (39). Other DR methods include the alteration of dietary structure, including changing or restricting the intake of some nutrient contents, as well as following a specific diet accurate to the exact percentage of every nutrient content (40). In preclinical and clinical research, different approaches are chosen according to specific requirements. For instance, preclinical research tends to adopt strategies that reduce the overall diet, or 24 h fasting every other day, to achieve DR. On the other hand, researchers usually have more flexible choices in clinical studies due to the adaptability of humans. In addition, in clinical studies, it is also possible to implement intermittent fasting (fasting on 2–3 days per week), intermittent energy restriction (reducing calorie intake on 4–6 days per week) or time restricted feeding (a restricted daily intake during a 4–6 h time window and fasting for the rest of the day) (41, 42). While benefiting health, DR has also been shown to extend lifespan in various experimental animals, including worms, flies, rodents, and primates (43). However, it is worth noting that in a chronic DR protocol (e.g., reduced daily food intake), a DR of 60% or above could be detrimental to lifespan even upon overall health conditions (1, 2). Interestingly, the advantages of DR are not universal when considered across species. Some studies showed that DR produces a longer lifespan and better health conditions in rats than in mice (44). Other studies, performed in primates, reported non-consistent results with regard to the effect of DR on lifespan (45, 46). These results may be due to the differences between species and DR protocols. Nevertheless, DR generally exerts beneficial effects on the extension of lifespan in various animal models. For a summary of common DR protocols mentioned above (see Table 1).

**Table 1:** A summary of common dietary restriction (DR) protocols.

DR protocol	Description	Key features
Caloric/Food Intake Reduction (39)	Reducing daily food or caloric intake (by 20–50% of ad libitum baseline) without changing the meal frequency.	The most extensively studied DR paradigm serves as a foundational reference for assessing DR effects.
Alternate-day Fasting (ADF) (39)	Alternating cycles of 24-h fasting and 24-h ad libitum feeding.	Induces stress resistance via periodic switching of feeding; may be better tolerated than continuous caloric restriction.
Intermittent Fasting (IF) (39)	Complete fasting (water only) for 2–3 days per week, with normal intake on other days.	A practical and sustainable protocol for human subjects.
Time-Restricted Feeding (TRF) (41)	Consuming all daily food within a consistent, narrow window (e.g., 4–6 h) and fasting for the remaining hours.	Focuses on the timing of intake rather than content/quantity.
Macronutrient Restriction (40)	Altering or restricting the proportion of specific macronutrients (e.g., low-protein, low-carbohydrate diets, ketogenic diet, Mediterranean diet).	Aims to investigate the role of specific nutrients; protocols require careful design to avoid malnutrition.
Formulated Diets (40)	Following specific diets with exact percentages of every nutrient.	Enables absolute control over nutrient composition, eliminating confounders from food sources.

### 2.3 Mechanisms underlying the mediatory effect of DR on inflammatory response

While neuroinflammatory processes may appear superficially self-contained, they intricately link pathogenic triggers to the mechanistic drivers underpinning neurodegenerative progression. When the brain is exposed to primary insults, such as traumatic injury or the aggregation of pathological proteins, the innate immune system becomes activated, eliciting a neuroinflammatory response to protect the CNS from detrimental stimuli. However, this process can be deleterious to the brain when accompanied by senescent degeneration (47). Cellular senescence represents an evolutionarily conserved tumor-suppressive program that halts the replication of genomically compromised cells through stable cell cycle arrest, thereby preventing malignant transformation. Paradoxically, this protective mechanism concomitantly activates the hypersecretion of pleiotropic factors, including metalloproteinases, proinflammatory interleukins, and chemotactic signals, collectively referred to as the senescence-associated secretory phenotype (SASP). SASP creates a chronic inflammatory environment that contributes to the progression of age-related neurodegenerative diseases (48, 49). Preclinical studies across diverse animal models and longitudinal human trials have demonstrated that sustained DR regimens consistently attenuate senescence-associated biomarkers in metabolically active tissues (50, 51).

The most obvious impact of DR is the reduction of adiposity. Adipose tissue functions as a dynamic endocrine organ, secreting a spectrum of immune-metabolic regulators, termed adipokines (e.g., leptin, adiponectin), that orchestrate systemic energy homeostasis and inflammation. Dysregulated adipokine secretion, particularly from hypertrophic visceral adipose tissue (VAT), directly contributes to systemic insulin resistance by impairing glucose uptake in skeletal muscle and hepatic insulin signaling (52). Insulin resistance and metabolic disturbance also represent the factors that could facilitate the progression of the inflammatory response (53). DR preferentially depletes VAT depots, the primary source of proinflammatory adipokines (TNF-α, IL-6) and free fatty acids that exacerbate lipid-induced insulin resistance in adipose tissues (54). This metabolic remodeling could shift adipose secretome profiles from pro- to anti-inflammatory polarization. Therefore, DR can ameliorate adiposity-associated insulin resistance by attenuating adipose tissue inflammation and by restoring systemic metabolic flexibility.

Beyond traditional pathophysiological frameworks, emerging evidence highlights the critical involvement of modifiable risk factors, including psychosocial stress, environmental neurotoxins, and lifestyle patterns, in priming neuroinflammatory states. Neuroinflammation can also be induced or even potentiated by peripheral inflammatory factors that could penetrate the BBB and reach the CNS, leading to the activation of microglia and astrocytes (55, 56). Over recent years, the prominent role of the gut-brain axis has been confirmed as a bidirectional connection in the communication between the peripheral system and CNS (57). The gut microbiota plays a primary role in this complex cross-talk. The gut microbiota has been found to exert clear functional effects on neural, endocrine, and immune mechanisms, in a manner that is mediated by the vagus nerve and the parasympathetic nervous system (58, 59). Emerging evidence also links DR to remodeling of the gut microbiota to influence neuroinflammation via the brain-gut axis. Dysbiosis increases intestinal permeability, allowing endotoxins (e.g., lipopolysaccharide, LPS) to enter systemic circulation and trigger peripheral inflammation. DR (including calorie restriction and intermittent fasting) is known to enrich beneficial taxa (e.g., Lactobacillus and Firmicutes) and increase the production of short-chain fatty acids while reducing the serum levels of LPS-binding protein, thus strengthening the intestinal barrier, inhibiting microglial activation, and reducing the production of proinflammatory cytokines (60, 61). Because DR can modulate the composition of the microbiota and induce metabolic and molecular adaptations at the whole-body level, including the brain, it is clearly possible that DR could also exert neuroprotective effects (62, 63).

On the other hand, neuroinflammatory responses are modulated by a network of molecular and cellular mechanisms operating at the micro-level. As a multifunctional mediator, NF-κB is found abundantly in neurons and glial cells (64). The persistent activation of NF-κB has been detected in many neurological diseases, including AD (65), PD (66), ALS (67), stroke (68), and other neurological diseases, due to its ability to regulate CNS development, cognitive function, neuronal survival, and neural plasticity (69–71). SASP components can also be regulated through the NF-κB signaling axis in aged or chronic inflammatory microenvironments (72). However, NF-κB-related neuroinflammatory reactions do not operate in isolation. In the aging brain, defective mitochondria and oxidative stress induced by mitochondrial dysfunction are known to contribute to the inflammatory process (73, 74). An increased mutational load in mitochondrial DNA (mtDNA) has been shown to result in malfunction of the mitochondrial electron transport chain (ETC), which can cause the release of endogenous reactive oxidant species (ROS), further exacerbating the inflammatory reaction (75, 76). Evidence has also shown that DR could induce mitophagy to promote the clearance of dysfunctional mitochondrial proteins and improve the age-related functional decline of ETC in mitochondria. Donato et al. also demonstrated that DR could reduce oxidative stress (60). Therefore, the improvement of mitochondrial dysfunction and oxidative stress brought about by the appropriate adoption of DR might represent another explanation for its anti-inflammatory effects. Other mechanisms have been suggested to be responsible for the neuroprotective mechanisms of DR, including the production of neurotrophic factors, cytoprotective protein chaperones, and the regulation of calcium (77–79).

## 3 Effects of DR on neurodegenerative diseases

### 3.1 Alzheimer’s disease

The AD is one of the most prevalent neurodegenerative diseases and is characterized by progressive cognitive decline and memory loss. Approximately 60–70% of all global dementia cases can be attributed to AD. Although the specific pathology of AD still remains unclear, neuroinflammation is suggested to be a potential underlying mechanism, alongside amyloid beta plaques and the accumulation of phosphorylated tau (80). Other than conventional therapeutic interventions, some authors have suggested that appropriate changes in lifestyle, such as calorie or DR, could be considered as an adjuvant therapeutic method.

#### 3.1.1 Preclinical studies of AD

Mutations in the genes encoding amyloid precursor protein (APP), presenilin 1 (PS1), and presenilin 2 (PS2) are responsible for most cases of familial AD (81). Thus, transgenic mice carrying mutations of these genes have been employed in several preclinical studies. Evidence from different research teams showed that long-term and short-term DR could reduce the levels of abnormal amyloid protein and improve cognitive function in transgenic mice carrying mutations in familial amyloid precursor protein (82–84). In two other studies using APP mutations, or *APP* and *PS1* double transgenic mice, as a model of AD, the number of Aβ plaques was significantly reduced in the hippocampus and cerebral cortex of the brain after a 40% restriction in calories (82, 85). Another study reported Aβ reduction as well as the improvement of cognitive function when intermittent fasting was utilized in 5-month-old APP/PS1 double transgenic mice (86). Scientists also obtained substantial evidence in 3xTg mice carrying mutations (*APPK670N/M671L* Swedish, *MAPT P301L*, and *PSEN1 M146V*) of familial AD. Different methods of DR (calorie restriction and intermittent fasting) were also confirmed to improve cognitive dysfunction and ameliorate Aβ and tau pathology (87). With regards to the modulation of molecular mechanisms, intermittent fasting was found to regulate GSK-3β through the interaction of multiple pathways (AMPK, BDNF, and PI3K-Akt pathways) to enhance neuronal differentiation and survival, as well as improve cognition in 3xTg-AD mice (88). Since Aβ exerts explicit adverse effects on synaptic function and because Guo and Mattson (89) proved that DR mimetics could resist the impairment of synaptic functionality induced by the deposition of Aβ in the rat, it is possible that long-term DR could exert protective effects on synaptic damage. Schafer et al. also reported that a 30% reduction in calories decreased the deposition of Aβ plaques in Tg2576 mice (*APPK670N/M671L* Swedish mutation) (83). These results were consistent with Wu et al., who suggested 30% calorie restriction could be beneficial to memory deficit and inflammatory reactive genes on conditional double knockout PS1 and PS2 mice (90). This evidence all suggested that different kinds of DR could be beneficial to the Aβ-associated pathology in rodent models.

As another Alzheimer-like neuropathological change, neurofibrillary tangles (NTF) are usually formed by hyperphosphorylated tau proteins (91). Brownlow et al. (92) found that calorie restriction for 3 months alleviated cognitive decline in Tg4510 (*TauP301L* mutation) mice. Furthermore, DR also exerted positive effects on pathological tau progression in 3xTg-AD, *PS1*, and *PS2* double knockout (cDKO) mice (87, 93, 94).

Although a neuroinflammatory response can be induced by abnormal Aβ and tau deposition, this response can also occur in the early stages of AD onset (95). Thus, neuroinflammation could be one of the factors that contribute to the development of AD. Compelling evidence showed that a dietary cycle that mimicked fasting improved cognitive behavior, reduced Aβ and tau load, while increasing hippocampal neurogenesis markers in 3xTg-AD mice and E4FAD mice (which express the human ApoE4 isoform). Moreover, under this DR system, the alleviation of microglia activation and reduced neuroinflammatory cytokines (e.g., TNF-α) were detected in the brains of model mice (94). Further evidence revealed an increase in the levels of neuroinflammatory markers in PS19 mice (*MAPTP301S PS19* mutation), which could be suppressed by an immunosuppressant (96). However, Lazic et al. (97) found that the expression of pro-inflammatory cytokines and Iba-1 was upregulated in 5XFAD mice (*APP K670_M671delinsNL* Swedish, APP I716V Florida, *APP V717I London*, *PSEN1 M146L*, *PSEN1 L286V* mutation) after alternative-day fasting for 4 months. Although the authors stated that the reasons for these negative effects needed more investigation, they assumed that the young age of the model mice (2 months-of-age) might represent one causative factor (97). It is noteworthy that calorie restriction did not exert beneficial effects on pathological changes such as amyloidosis in the prefrontal cortex of extremely aged rhesus macaques (22–44 years old, average age 31.8 years) (98).

#### 3.1.2 Clinical studies on AD

From an epidemiological perspective, obesity has been shown to be partially responsible for an increased risk of dementia. In an average 21-year follow-up cohort study, convincing data revealed a cohesive relationship between midlife obesity and the risk of AD (99). Due to the strong link between DR and obesity, it is of great significance to conduct in-depth investigations of the specific relationship between obesity, dementia, and DR. In a 12-month, single-center, prospective, and controlled clinical trial featuring 80 obese patients with mild cognitive impairment, weight reduction was associated with an improvement in global cognition, verbal function, insulin resistance, as well as inflammatory response (100). In another study, Taylor et al. (101) investigated patients with mild AD; 10 out of 15 patients followed a ketogenic diet for 3 months (followed by 1 month of a regular diet) and showed an improvement in cognitive function. Another ongoing study includes 40 patients with amnestic mild cognitive impairment; preliminary data showed that a fasting-mimicking diet was safe and feasible; the effects of a fasting-mimicking diet on cognitive function will be analyzed when more data have been collected (94). Although positive evidence has been detected in several studies, the practical application of DR for AD patients still needs more profound investigation and validation. Table 2 shows a summary of all existing preclinical and clinical studies.

**Table 2:** All reviewed preclinical and clinical studies investigating the influence of DR in AD.

Model	DR method	Cognitive and behavioral function	Biomarkers	Brain pathology	Reference
AD preclinical studies					
(a) APP (J20) mice (b) APPandPS1 mice	(a) 40% CR, 6 weeks (b) 40% CR, 14 weeks	N/A	↓ GFAP	↓ Aβ plaque	Patel et al. (2005) (82)
Tg2576 mice	30% CR, 2.8 or 12.5 months	N/A	N/A	↓ Aβ plaque ↓ Aβ level ↓ Psenen, Ps1 γ-secretase subunit transcript levels	Schafer, et al. (2015) (83)
Tg2576 mice	30% CR, 9 months	N/A	N/A	↓ Aβ plaque ↓ Aβ level	Wang et al. (2005) (84)
APP/PS1 mice	40% CR, 14 weeks	N/A	N/A	↓ Aβ deposits	Mouton et al. (2009) (85)
APP/PS1 [B6C3-Tg (APPswe, PS1dE) 85Dbo/J] mice	ADF, 5 months	↑ Cognitive function	N/A	↓ Aβ plaque	Zhang et al. (2017) (86)
3xTg AD mice	40% CR or ADF,14 months	↑ Cognitive function	N/A	↓ Aβ level ↓ phospho-tau (40% CR group)	Halagappa et al. (2007) (87)
3xTg AD mice	IF, 3 months	↑ Cognitive function	↑ GSK-3β ↑ AMPK ↑ BDNF ↓ Insulin	↑ Neuronal differentiation	Li et al. (2020) (88)
PS1 and PS2 conditional double knockout mice	30% CR, 4 months	↑ Cognitive function	↓ GFAP; ↓ Cleaved caspase3 ↓ inflammatory genes	↓ phospho-tau	Wu et al. (2008) (90)
Tg4510 mice	CR, 3 months (lost 35% bodyweight)	↑ Cognitive function	N/A	N/A	Brownlow et al. (2014) (92)
3xTg AD mice	PRC, 18–19 weeks	↑ Cognitive function	↓ IGF-1 levels, ↑ IGFBP-1, n.s. CD11b	↓ phospho-tau n.s, Aβ plaque	Parrella et al. (2013) (93)
(a) E4FAD mice (b) 3xTg AD mice	(a) FMD, 4.5 months (b) FMD or PR, 15.5 months; FMD, 2 months	(a) and (b)↑ Cognitive function	(a) NOX2 levels ↓ proinflammatory cytokines (b) ↓ NOX2 levels, ↓ Iba-1 ↓ proinflammatory cytokines	(a) ↓ Aβ plaques ↓ Aβ levels (b) ↓ Aβ plaques, ↓phospho-tau	Rangan et al. (2022) (94)
5XFAD mice	EOD, 4 months	n.s.	↑ Iba-1 ↑ GFAP ↑ proinflammatory cytokine	n.s., Aβ accumulation	Lazic et al. (2020) (97)

Patients population	Age and sex	Dietary interventions	Results	Reference
AD clinical studies				
Adults with obesity and MCI	Older than 60 Female and male	CR with nutritional counseling, 12 months	↑ global cognitive function ↑ memory ↑ semantic fluency	Horie et al. (2016) (100)
Adults with very mild, mild, moderate AD	Mean age 78 Female and male	MCT-supplemented KD, 3 months	↑ cognitive function	Taylor et al. (2017) (101)
Adults with aMCI or mild AD	Age 55–80 female and male	FMD cycles every other month, 12 months	Feasible and safe N/A, cognitive function (lack of data)	Rangan et al. (2022) (94)

CR, calorie restriction; ADF, alternative-day fasting; IF, intermittent fasting; PRC, protein restriction cycle; FMD, fasting-mimicking diet; EOD, every-other-day feeding; MCT, medium chain triglyceride; KD, ketogenic diet; aMCI, amnestic mild cognitive impairment; N/A, data not available; n.s, data has no significance.

### 3.2 Parkinson’s disease

The PD is the second most common neurodegenerative disease in the elderly population. The symptoms of PD are characterized by progressive, unintended, or uncontrollable movements (including resting tremor, stiffness, bradykinesia, difficulty with balance and coordination), which begin gradually and worsen over time (102). The identified putative mechanisms of PD include the accumulation of *α*-synuclein (Lewy Bodies) and dysfunction of the dopaminergic neurons in the substantia nigra (SN); these symptoms are associated with multiple underlying molecular mechanisms, including oxidative stress, neuroinflammation, and mitochondrial dysfunction. For instance, activation of microglia can be induced by the accumulation of α-synuclein and further result in inflammatory and oxidative damage in the CNS. In turn, the neuroinflammatory response can also promote the formation of misfolded α-synuclein, thus creating a vicious circle (103, 104).

#### 3.2.1 Preclinical studies of PD

Compelling preclinical data revealed a high correlation between DR and the development of PD. Evidence showed that obesity induced by a high-fat diet significantly compromised the normal functionality of dopaminergic neurons in the SN in a 1-methyl-4-phenyl-1,2,3,6-tetrahydropyridine (MPTP) induced mouse model of PD (105). Furthermore, Duan and Mattson (106) proved that applying 3 months of alternative-day fasting in C57BL/6 mice prior to MPTP injection could attenuate the loss of dopaminergic neurons in the SN and improve motor coordination in a rotarod test. Similar results were also evident in a study employing two cycles of a fast-mimicking diet before and one cycle of the same diet regimen after MPTP injection in C57BL/6 mice. The authors of this study also observed a reduction in microglial activation and a reduction in the expression of inflammatory cytokines (107). In another study, Griffioen et al. (108) used a genetic mouse model carrying a mutation of A53T *α*-synuclein to evaluate the effects of alternative-day fasting under a high-fat diet. Mice possessing the A53T α-synuclein mutation exhibited a dysfunctional autonomic nervous system accompanied by an impaired cardiovascular stress response, α-synuclein aggregation, and motor impairment. Analysis showed that alternative-day fasting improved abnormal functionality of the autonomic nervous system (108).

One hypothesis as to why DR might be associated with PD is the origination of α-synuclein from the gastrointestinal tract and vagus nerve transition. In this hypothetical scenario, Bayliss et al. (109) discovered that 30% calorie restriction for 27 days successfully restored the loss of dopaminergic neurons induced by MPTP administration in Wild-Type (WT) mice, although the same DR regimen failed to exert similar neuroprotective effects in ghrelin KO mice. In a WT group with calorie restriction, the authors detected a higher level of phosphorated AMPK expression, thus suggesting that AMPK could represent a potential target for the neuroprotective effects of ghrelin (109). Similar results were detected in a lactacystin (LAC) model, in that 30% calorie restriction protected against the loss of dopaminergic neurons by degradation. These effects were verified on ghrelin receptor KO mice and further indicated that the observed effects were independent of ghrelin. Collectively, these results suggested that DR might act as an adjuvant therapeutic method for PD (109, 110).

Research involving rhesus monkeys provided further evidence for the effects of DR. Maswood et al. (111) reduced the calorie intake to 30% prior to MPTP injection during a six-month experiment. Analysis revealed that 30% calorie restriction increased dopaminergic neurons, dopamine levels, and metabolites in the brains of rhesus monkeys following MPTP injection; glial cell line-derived neurotrophic factor (GDNF) was also upregulated in the 30% calorie restriction group (111).

However, it is worth noting that the effects of the DR regime have not always been satisfactory in all animal models of PD. For example, alternative day fasting prior to the administration of 6-hydroxy dopamine (6-OHDA) failed to rescue the loss of dopaminergic neurons in Sprague–Dawley rats (112). Compared to the evidence we summarized above, these results seem contradictory. However, the relatively short period of observation might be the reason why the neuroprotective effects of calorie restriction were not detected. Furthermore, the neurotoxic effects of 6-OHDA are far stronger than MPTP in terms of dopaminergic neuronal damage. Rotenone is another neurotoxin that can cause the degeneration of dopaminergic neurons in the SN. Coincidentally, alternative day fasting, along with rotenone treatment, was found to reduce TH + neurons (tyrosine hydroxylase positive, a marker of dopaminergic neurons) and an increase in the accumulation of *α*-synuclein. The authors assumed that these exacerbated effects of alternative-day fasting were caused by high levels of inflammatory phospholipids and excitatory amino acids in the SN area (113).

#### 3.2.2 Clinical study on PD

Due to the evidence of different DR regimes gained from preclinical studies, a few clinical trials have been conducted to evaluate the feasibility and efficacy of DR in PD patients. In a randomized controlled clinical trial, a ketogenic diet and a low-fat high- carbohydrate diet were found to improve motor and non-motor symptoms in patients who completed the whole diet process (114). Another clinical study enrolled seven patients to assess the feasibility of the hyper-ketogenic diet. In most patients, the unified PD score improved after completing 28-days of the hyper-ketogenic diet (115). However, thus far, evidence from clinical studies remains limited. It is important that the risks of DR (described later) are fully considered when further clinical studies are conducted. Table 3 provides a summary of all preclinical and clinical studies.

**Table 3:** All reviewed preclinical and clinical studies investigating the influence of DR in PD.

Model	DR method	Behavioral function	Biomarkers	Brain pathology	Reference
PD preclinical studies					
C57Bl/6 mice MPTP model	ADF, 3 months Before MPTP injection	↑ motor coordination	N/A	↑ SN dopaminergic neurons	Duan and Mattson (1999) (106)
C57Bl/6 mice MPTP model	FMD, 2 cycle before and 1 cycle after MPTP injection	↑ motor coordination	↓ TNF-α, IL-1β ↓ Iba-1 ↑ GFAP	↑ SN dopaminergic neurons	Zhou et al. (2019) (107)
THY1-SNCA*A53T mice	ADF, 12 weeks	↑ motor coordination	N/A	↓ ANS dysfunction	Griffioen et al. (2013) (108)
Ghrelin WT/KO mice MPTP model	30% CR, 27 days	n.s.	↑ phospho-AMPK ↑ ACC	↑ SN dopaminergic neurons	Bayliss et al. (2016) (109)
Ghrelin receptor WT/KO mice LAC model	30% CR, 28 days (CR 21 days – inject LAC – CR 7 days)	N/A	N/A	↑ SN dopaminergic neurons ↑ striatal dopamine levels	Coppens et al. (2017) (110)
Sprague–Dawley rats 6-OHDA, model	ADF, 2 or 8 weeks	N/A	N/A	n.s.	Armentero et al. (2008) (112)
C57BL/6J mice Rotenone model	ADF, 28 days	↓ motor coordination	↓ IGF-1 ↑ SM ↑ LPA, LPC	↓ SN dopaminergic neurons ↑ SN alpha-synuclein	Tatulli et al. (2018) (113)
Rhesus monkey MPTP model	30% CR, 6 months before MPTP injection	↑ motor coordination	↑ GDNF	↑ SN dopaminergic neurons ↑ striatal dopamine levels	Maswood et al. (2004) (111)

Patients population	Age and sex	Dietary interventions	Results	Reference
PD clinical studies				
Adults with PD	Age 40–75 Female and male	LFD or KD, 8 weeks	↓ DS-UPDRS scores ↑ motor and non-motor symptoms	Phillips et al. (2018) (114)
Obese adults or overweight adults with PD	Mean age 63 Female and male	HKD, 28 days	n.s.	Vanitallie et al. (2005) (115)

MPTP, 1-methyl-4-phenyl-1,2,3,6-tetrathydropyridine; SN, substantia nigra; ANS, autonomic nervous system; ACC: LAC, lactacystin; SM, sphingomyelin; LPA, lysophosphatidic acid; LPC, lysophosphatidylcholine; LFD, low-fat diet; HKD, hyperketogenic diet; N/A, data not available; n.s, data have no significance.

### 3.3 Multiple sclerosis

The MS is a neuronal demyelinating disease that causes axonal and neuronal damage. As an inflammatory disease, the primary pathological change of MS is myelin damage in neurons induced by the infiltration of peripheral immune cells into the CNS and spinal cord (116). After years of research, the precise mechanisms underlying MS remain unclear, although one hypothesis suggests that T-cell mediation plays an important role in the pathological progression of MS (117). However, other studies have shown that obesity and its complications represent a high-risk factor for the progression of MS. The severity of obesity could influence the response to therapy (118–120). In this case, how to introduce DR into the treatment of MS is worth more attention.

#### 3.3.1 Preclinical study on MS

In MS research, the most commonly used animal model is experimental autoimmune encephalomyelitis (EAE). To generate an EAE model, the animal is injected with an antigen (e.g., myelin oligodendrocyte glycoprotein) against the myelin sheath to induce demyelinating changes in the CNS (121). In several preclinical studies, different types of DR regimes were proven to reduce MS pathology. Using an EAE model, alternative day fasting (over a duration of 4–8 weeks) and calorie restriction (from 33 to 66%) were reported to reduce the clinical severity score as well as the incidence rate (122). Similar results were observed in studies employing the fasting-mimicking diet in EAE mice. Promising evidence demonstrated that, compared to the control-fed EAE mice group, the model mice showed an obvious reduction in the incidence of MS after three fast-mimicking cycles (123). In another study, two cycles of the fast-mimicking diet for three weeks also exhibited positive effects on symptoms (124). In the same EAE model, the administration of a ketogenic diet also exerted effective therapeutic activity. Analysis showed that the ketogenic diet not only reduced the clinical score but also prevented motor deficiency (125, 126).

In addition, the anti-inflammatory effects of DR were also proven in multiple preclinical studies. In a model of MS induced by Cuprizone, 10 and 33% calorie restriction prevented microglia activation. Moreover, calorie restriction mediated microglia polarization by increasing levels of the M2 phenotype marker arg-1 and decreasing levels of the M1 phenotype marker iNOS (127, 128). Sun et al. obtained similar results in that 24 days of a ketogenic diet shifted microglial polarization from the harmful M1 to protective M2 phenotype, along with downregulation of pro-inflammatory cytokines, including TNF-*α*, IL-1β, and IL-6 (125). Choi et al. (123) and Piccio et al. (129) reported that the fast-mimicking diet and 40% calorie restriction could increase the level of corticosterone and reduce the levels of pro-inflammatory cytokines simultaneously (123, 129). Due to the intimate relationship between autoimmune reaction and inflammatory response, several studies provide adequate evidence for the mediatory effects of DR in immunoreaction. In two successive studies on EAE mice conducted by Sun et al. and Zhang et al., 24 days of a ketogenic diet downregulated the levels of CXCR3, CXCL10, CCR5, CCR1, CCR2, CCL3, and CCL2 in the spinal cord and spleen, and prevented the infiltration of T-cells and monocytes into the CNS (125, 126). In another study, Esquifino et al. (122) reported that 15 days of 66% calorie restriction reduced the mitogenic response induced by a T-cell activator, concanavalin, in a Lewis rat model of EAE. In addition, alternative-day fasting showed an explicit ability to attenuate the autoimmune response by influencing the inflammatory monocyte pool, resulting in a reduction in the expression of pro-inflammatory genes, including *CXCL2* and *CXCL10*, in the spinal cord (130). Furthermore, Choi et al. and Bai et al. reported that a fast-mimicking diet promoted oligodendrocyte regeneration in the spinal cord of EAE mice. A vibrant regeneration and differentiation of oligodendrocytes was observed in EAE mice after three cycles of the fast-mimicking diet. Similarly, two cycles of a fast-mimicking diet regime at 3-weeks post-induction of EAE led to the increased proliferation of oligodendrocytes (123, 124).

#### 3.3.2 Clinical studies of MS

Multiple clinical studies have evaluated and confirmed the safety and feasibility of DR intervention in MS patients. For instance, Cozart et al. (131) reported that a 24-week weight loss program could successfully help patients with obesity and MS to achieve clinically meaningful weight loss without compromising their health. Obesity is a risk factor for the development of MS; a recent randomized, controlled clinical study by Bruce et al. enrolled 71 patients with MS who were randomized to a weight loss intervention or treatment-as-usual groups. Analysis showed that 65% of patients in the weight loss intervention group successfully achieved clinically meaningful weight loss (≥5% of their original weight). Furthermore, weight loss was associated with MS-related symptoms, including improved mobility and the alleviation of fatigue (132). In another randomized, controlled trial, Chase et al. recruited 39 patients with MS and showed that a low-fat diet reduced calorie intake and improved fatigue (133). Cignarella et al. (134) designed a 15-day alternative-day fasting regimen and enrolled patients with acute MS relapse in a single-center randomized controlled pilot clinical trial. Results showed a decreased serum leptin and a reduction of T-cells and B-cells in the blood of patients in the fasting group, which recapitulated their previous animal studies (134). Similar results were also reported by Fitzgerald et al. (135) in that intermittent fasting could induce a significant modification of T-cells in patients with MS. These findings of reduced memory T-cells in MS patients with intermittent fasting are consistent with those published by Choi et al. (123) and Cignarella et al. (134) in EAE mice with a fasting-mimic diet. These findings showed that DR made the predominant contribution to weight loss; however, the actual clinical effects of DR on MS still need further investigation. Table 4 provides a summary of all preclinical and clinical studies on DR and MS.

**Table 4:** All reviewed preclinical and clinical studies investigating the influence of DR in MS.

Model	DR method	Clinical score and pathological changes	Immune findings and biomarkers	Reference
MS preclinical studies				
Lewis rats EAE model (SCH)	66% or 33% CR, 15 days pre-induction	↓ clinical score	↑ immunological status ↓ IFN-γ in spleen and lymph	Esquifino et al. (2007) (122)
C57BL/6 mice EAE model (MOG)	FMD, 3 cycles after 10% or 100% showed EAE signs	↓ clinical score (reversed symptoms in 20%) ↓ demyelination	↓ spinal cord inflammation ↓ CD4 and CD8 T-cells in the CNS ↓ lymphocyte, monocytes	Choi et al. (2016) (123)
C57BL/6 mice EAE model (MOG)	FMD, 2 cycles, at 3 weeks post-induction	↓ clinical score ↓ demyelination ↑ myelin regeneration	↓ spinal cord inflammation ↑ CD4 + T cells and INF-*γ*-producing Th1 cells ↑ BDNF	Bai et al. (2021) (124)
C57BL/6 mice EAE model (MOG)	KD, 24 days with induction	↓ clinical score ↑ motor coordination ↓ demyelination	↓ spinal cord inflammation ↓ TNF-α, IL-1β and IL-6 ↓ CCL2, CCR2, CCL3, CCR1, CCR5, CXCL10, and CXCR3 ↓ NLRP3 and Iba-1 ↑TGF-β	Sun et al. (2023) (125)
C57BL/6 mice EAE model (MOG)	KD, 24 days with induction	↓ demyelination ↑ myelin regeneration	↓ spinal cord inflammation ↓ ratios of Th1/Th2 and Th17/Treg ↓ T-bet, IFN-γ, RORγt, and IL-17 ↑GATA3, IL-4, Foxp3, and IL-10	Zhang et al. (2024) (126)
C57BL/6 mice CPZ demyelination model	33% CR, 4 weeks at 12 weeks post-induction	↑ motor coordination ↑ balance performance ↓ demyelination	↓ astrogliosis and microgliosis ↑ BDNF and Sox2	Mojaverrostami et al. (2020) (127)
C57BL/6 mice CPZ demyelination model	CR, 6 weeks, a diet mixed with 10% CMC and CPZ	↑ motor coordination ↑ balance performance ↓ demyelination	↓ iNOS, TNF-*α*, Iba-1 ↑ Arg-1	Zarini et al. (2021) (128)
SJL mice EAE model (PLP)	40% CR, 5 weeks pre-induction	↓ clinical score ↑ survival ↓ demyelination ↓ axon damage	↓ spinal cord inflammation ↓ leptin and IL-6 ↑ adiponectin and corticosterone	Picco et al. (2008) (129)
C57BL/6 mice EAE model (MOG)	ADF, 4 weeks pre-induction	↓ clinical score	↓ inflammatory monocytes ↓ TNFα, IL-1β, CXCL2 and CXCL10	Jordan et al. (2019) (130)

Patients population	Age and sex	Dietary interventions	Results	Reference
MS clinical studies				
Adults with RRMS patients	18–68 age Female and male	FMD 1 cycle followed by a Mediterranean diet or KD, 6 months	↑ HRQOL (in FMD and KD group)	Choi et al. (2016) (123)
Adults with MS and obesity	18–70 age Female and male	CR (1200–1,500 kcal per day) and exercise 46.2 min per week, 6 months	↑ mobility ↓ fatigability ↑ quality of life	Bruce et al. (2023) (132)
Adults with MS	18–70 age Female and male	CR (no more than 20% fat intake), 16 weeks	↓ fatigability	Chase et al. (2023) (133)
Adult RRMS patients during relapse	18–60 age Female and male	ADF, 15 days	↓ blood T and B lymphocytes ↓ naïve CD4 + cells ↓ Leptin	Cignarella et al. (2018) (134)
Adult RRMS patients	18–50 age Female and male	22% CR daily or 75% CR 2 days per week, 8 weeks	↓ effector memory ↓ memory T cells ↑ naïve T cells	Fitzgerald et al. (2022) (135)

EAE, experimental allergic encephalomyelitis; SCH, spinal cord homogenate; CPZ, Cuprizone; CMC, carboxymethyl cellulose; RRMS, relapsing remitting multiple sclerosis; HRQOL, health-related quality of life; N/A, data not available; n.s, data have no significance.

### 3.4 Amyotrophic lateral sclerosis

The ALS is a neurodegenerative disorder caused by the degradation of the upper and lower motor neurons. The degeneration of motor neurons leads to the denervation of voluntary muscles, ultimately resulting in progressive muscle paralysis. Over 80% of ALS cases are sporadic, while the remaining cases are classified as familial, one-third of which possess mutations in Cu/Zn superoxide dismutase 1 (SOD1). Although animal models (mostly rodents) possessing mutations in SOD1 cannot typically represent ALS cases, this genetically mutated model is still used in pharmacology screening research due to its motor neuron degeneration and muscle atrophy phenotype (136, 137). As the in-depth study progresses, new genetic rodent models with broader relevance have been developed, such as the genetic mutation in *TDP-43*, *FUS*, and *C9orf72* (138).

#### 3.4.1 Preclinical and clinical studies of ALS

Although DR is supposed to be beneficial to lifespan and neuroinflammation, existing data can provide conflicting information. Critically, current preclinical evidence does not support DR as a therapeutic strategy for ALS. Evidence provided by Pedersen and Mattson indicated that, compared to mice fed ad libitum, DR did not influence disease onset and shortened disease duration. These results suggested that DR accelerated the clinical course and provided no benefits to a mouse model exhibiting SOD1 mutations (139). In two successive studies conducted by Hamadeh et al. (140) and Hamadeh and Tarnopolsky (141), the authors concluded that DR might not be a protective therapeutic strategy for patients with ALS due to the hastening effects of short-term and long-term DR on the clinical onset of disease in the *SOD1^G93A^* mice model. With regards to the potential mechanisms underlying these adverse effects, Petel et al. provided evidence that DR weakened mitochondrial bioenergetic efficiency and damaged the stress response, thus resulting in increased lipoperoxidation, inflammation, and apoptosis (142, 143). To the best of our knowledge, there has been no clinical trial investigating how DR can influence ALS. Therefore, based on available preclinical data, DR should not be considered as a viable neuroprotective intervention for ALS. Table 5 shows a summary of all preclinical and clinical studies relating to DR and ALS.

**Table 5:** All reviewed preclinical and clinical studies investigating the influence of DR in ALS.

Model	DR method	Motor function and pathological changes	Biomarkers and other findings	Reference
ALS preclinical studies				
G93A mice	60% CR, around 130 days	↑ motor function	↑ time of clinical onset	Hamadeh et al. (2004) (140)
G93A mice	60% CR, around 130 days	↑ motor function	↑ time of reaching disease endpoint	Hamadeh and Tarnopolsky (2006) (141)
G93A mice	60% CR, around 135 days	↓ motor function	↑ lipid peroxidation ↑ inflammation ↓ protein oxidation and stress response	Patel et al. (2010) (142)
G93A mice	60% CR, around 135 days	↓ motor function	↑ apoptosis ↓ cell stress response	Patel et al. (2009) (143)

### 3.5 Huntington’s disease

Huntington’s disease (HD) is a devastating and inherited autosomal-dominant neurodegenerative disease. The distinct phenotypes of HD include abnormal movement control (most commonly seen as chorea and dystonia), incoordination, cognitive decline, and psychiatric disorders. The aggregation of mutated huntingtin protein (encoded by an expanded triplet CAG repeat in the Huntingtin gene) is considered to be the cause of neuronal dysfunction in HD. Furthermore, various cellular pathways and related functional proteins are involved in the progression of neuronal degradation, resulting in oxidative stress, excitotoxicity, inflammatory reaction, and bioenergetic deficiencies (144).

#### 3.5.1 Preclinical and clinical studies on HD

Studies have shown that different DR regimes could provide benefits for the progression of HD. In preclinical studies, several transgenic mouse models of HD have been designed and utilized. For example, Duan et al. (145) conducted alternative-day fasting on a *N171-82Q* transgenic mouse model of HD. Analysis revealed that alternative-day fasting improved motor function and delayed disease onset while the accumulation of mutated huntingtin protein decreased; this was accompanied by a reduction in brain atrophy (145). Wang et al. (146) and Whittaker et al. (147) found that circadian locomotor activity and coordination in the onset of sleep were increased following 3 months of time-restricted eating in *Q175 HD* and *BACHD* transgenic mice. Another study utilized *YAC128 HD* transgenic mice with one-week time-restricted eating, evidence indicated that the accumulation of cortical mutated huntingtin protein decreased significantly, perhaps due to the enhancement of neuronal autophagy (147). Although there is a lack of data from clinical trials, Phillips et al. published a case report describing the positive effects of a time-restricted ketogenic diet on a 41-year-old patient with HD. In this article, improvements of motor symptoms, composite unified HD rating scale score, behavioral problems, irritability, and mood-related quality-of-life were observed; no adverse effects were reported (148). Table 6 shows a summary of all preclinical and clinical studies of DR and HD.

**Table 6:** All reviewed preclinical and clinical studies investigating the influence of DR in HD.

Model	DR method	Motor function and Pathological changes	Biomarkers and other findings	Reference
HD preclinical studies				
HD-N171-82Q (+/−, HD) transgenic mice	ADF, 3 months	↑ motor function ↓ brain aggregate formation	↓ blood glucose ↓ caspase-1 ↑ BDNF, HSP-70	Duan et al. (2003) (145)
Q175 transgenic mice	TRF, 3 months	↑ motor function ↑ sleep	↑ heart rate variability	Wang et al. (2018) (146)
BACHD mice model	TRF, 3 months	↑ motor function ↑ sleep	↑ heart rate variability	Whittaker et al. (2018) (147)

Patients population	Age and sex	Dietary interventions	Results	Reference
HD clinical studies				
One patient with progressive, deteriorating HD	41-year-old	TRKD, 48 weeks	↑ motor symptoms ↑ daily activities ↑ cUHDRS ↑ apathy, disorientation, anger, and irritability	Phillips et al. (2022) (148)

TRF, time restricted feeding; TRKD, time restricted ketogenic diet.

## 4 DR and other neurological disorders

### 4.1 Epilepsy

Epilepsy is a chronic brain disorder that causes recurring and unprovoked seizures. Abnormal intracerebral electrical signals are considered to be the apparent cause that induces seizures, yet the specific underlying mechanisms of epilepsy remain unclear after years of research. Thus, there is no known cause in over 40% of epilepsy patients; consequently, more than one-third of patients experience no response to existing drugs (known as drug-resistant epilepsy, DRE) (149). Consequently, there is an urgent need to identify alternative or complementary therapeutic methods. As early as the ancient Greek period, Hippocrates identified that dietary restriction, such as fasting, could attenuate the symptoms of epilepsy. Prior to the 1900s, fasting was reported as the predominant therapeutic method; however, since this time, fasting has been increasingly replaced by the ketogenic diet in clinical trials (150–152).

#### 4.1.1 Preclinical studies of epilepsy

Rodent models have been developed to investigate the effect of DR on epilepsy. For instance, the lithium-pilocarpine Wistar rat model showed a reduction in seizure severity score and forelimb clonus seizure in a time-restricted feeding group. The anticonvulsant effects of time-restricted feeding were also reflected in the late phase of electroencephalography recordings. The authors suggested that the Akt-AMPK pathway could be involved in the effects induced by time-restricted feeding (153). In another study, 15% calorie restriction increased the levels of phosphorylated AMPK and further resulted in a reduction of ribosomal protein S6, thus influencing the mTOR cascade. These results suggested that inhibition of the AMPK-related mTOR cascade could represent a mechanism responsible for the effects of calorie restriction (154). Two studies conducted by Yuskaitis et al. (155, 156) indicated that seizures might represent a DEPDC5-dependent phenomenon. In these studies, Depdc5cc + mice (conditional knockout of DEPDC5 exclusively in neurons) were administered with pentylenetetrazol (PTZ) to induce seizure-like symptoms. Analysis showed that 24-h fasting did not change the incidence of seizures caused by the absence of the DEPDC5 gene. Furthermore, Yuskaitis et al. (155, 156) provided proof that amino acid-associated mTOR elevation was dependent on the presence of DEPDC5.

Since the ketogenic diet is considered to exert positive effects in the therapeutic process for epilepsy, researchers began to focus on comparative studies between the ketogenic diet and other forms of DR. Eagles et al. (157) found that in 5-week Sprague–Dawley rats, 50% calorie restriction of a high-carbohydrate diet could increase the seizure threshold induced by PTZ and that this effect was almost equal to rats fed with a ketogenic diet. However, Hartman et al. (158) did not evaluate the seizure threshold in HIH Swiss mice with intermittent fasting under a PTZ model. When compared to a ketogenic diet, intermittent fasting was protective against kainic acid, whereas the ketogenic diet failed to provide protection. Conversely, the ketogenic diet had positive effects on the seizure symptoms induced by 6 Hz, and intermittent fasting increased the activity of seizures (158). In a maximal dentate activation model of epileptogenesis, Bough et al. (159) found that ketogenic calorie-restricted animals showed a remarkable reduction in the duration of electrographic seizures when compared to normal calorie-restricted animals. Greene et al. (160, 161) chose EL mice (a seizure-prone genetic model) to investigate the effects of a ketogenic diet and calorie restriction. Interestingly, 15 and 30% calorie restriction exerted a better antiepileptogenic effect than a ketogenic diet; however, when calorie restriction was adjusted according to body weight, the anticonvulsive effects of calorie restriction and the ketogenic diet became homogeneous (160–162). Although similar effects have been identified in several preclinical studies, existing evidence indicates that the anticonvulsive effects of a ketogenic diet and other forms of DR could be very different.

With regards to the underlying mechanisms, the ketogenic diet was found to elevate the levels of β-hydroxybutyrate and reduce the levels of blood glucose during the prevention of seizures. Greene et al. (160, 161) suggested that plasma glucose was closely related to seizure susceptibility in EL mice. Furthermore, β-hydroxybutyrate was found to be inversely correlated with seizure severity score in a rat pilocarpine model (160, 161). It is worth noting that neuroinflammation has been proven to play an important role in the progression of epilepsy. Studies of different models *in vivo* and *in vitro* have provided abundant proof for increased inflammatory cytokine levels and the modification of associated pathways (163). One study used LPS to mimic the inflammatory environment in vitro; the authors identified that the neuroinflammatory reaction could be attenuated by β-hydroxybutyrate in microglia cells (164). Preclinical evidence for the neuroinflammatory response in the pathogenesis of epilepsy and the positive effects of the ketogenic diet on neuroinflammation indicate the need for further research in the context of epilepsy.

#### 4.1.2 Clinical study on epilepsy

When investigating clinical studies of epilepsy, we found that most existing data were derived from children. Epilepsy is an age-related neurological disease, and adults (especially those over 60 years of age) tend to be more susceptible to this disease. Thus, existing references provide very little clinical data relating to adults in the area of DR treatment and epilepsy. The only randomized clinical study in adults was conducted by Zare et al. (165), who applied a modified Atkins diet (a type of ketogenic diet with less restriction) and enrolled 54 patients with epilepsy. Analysis showed that 2 months of a modified Atkins diet successfully reduced the frequency of seizures (165). Bergqvist et al. (166) enrolled male and female children with DRE and showed that 24–48 h of fasting before 3 months of a ketogenic diet clearly reduced the frequency of seizures. Similar results were reported in some other randomized clinical trials using ketogenic diets, along with verification of the applicability and safety of ketogenic diets (167, 168). Moreover, the anti-inflammatory effects of ketogenic diets were also identified in patients with epilepsy; the levels of inflammatory cytokines (CXCL13 and IL-6 in adult patients; CCL21 and CCL27 in pediatric patients) were significantly reduced (169, 170). Table 7 provides a summary of all preclinical and clinical studies referring to diets and epilepsy.

**Table 7:** All reviewed preclinical and clinical studies investigating the influence of DR in epilepsy.

Model	DR method	Seizure induction	Changes of seizure symptoms and biomarkers	Reference
Epilepsy preclinical studies				
Wistar rats	TRF (2 h per day), 20 days	Lithium-pilocarpine model	↓ seizure severity score ↑ seizure latency ↓ late phase EEG power	Landgrave-Gomez et al. (2016) (153)
Wistar rats	15% CR, 30 days	Amygdala electrical kindling	↓ after-discharge threshold and duration	Phillips-Farfán et al. (2015) (154)
Depdc5 conditional knockout mice (*Depdc5cc^+^*) Littermate control mice	24 h of fasting	Acute PTZ seizure model	↑ seizure symptom (*Depdc5cc^+^* mice) ↓ seizure symptom (littermate mice)	Yuskaitis et al. (2022) (155)
Sprague–Dawley rats	50, 35, 10% CR or KD, 20 days	PTZ Administered Until seizure	↑ seizure threshold (KD group = 50% CR group)	Eagles et al. (2003) (157)
NIH Swiss mice	ADF or KD, 12 days	6 Hz test and MES PTZ and KA	KD: ↑ 6 Hz and MES threshold, ADF: ↓ KA seizure, ↓ 6 Hz, and MES threshold	Hartman et al. (2010) (158)
Sprague–Dawley rats	KD with 15%CR and 15% CR, 28 days	Hippocampal electrophysiology, I/O, paired pulse, MDA	CR: ↓ PS amplitude and SD events, MDA ↑ threshold, and ↑ latency KD (with 15% CR): ↓ PS amplitude and SD events, ↑ MDA threshold, and ↓ latency	Bough et al. (2003) (159)
EL mice	30, 15%CR, or KD, 10 weeks	Handling induced stress	↓ onset and incidence of seizures	Greene et al. (2001) (160)

Patients population	Age and sex	Dietary interventions	Results	Reference
Epilepsy clinical studies				
Adult with refractory epilepsy	18–57 age Female and male	AD or mAD, 2 months	n.s.	Zare et al. (2017) (165)
Children with intractable epilepsy	1–14 age Female and male	FAST-KD or GRAD-KD, 3 months	FAST-KD: ↓ seizure frequency, ↓ time to ketosis GRAD-KD: ↓ seizure frequency	Bergqvist et al. (2005) (166)
Children with drug-resistant epilepsy	1–15 age Female and male	KD, MAD, or LGIT diet, 24 weeks	↓ seizure frequency in all three groups	Sondhi et al. (2020) (167)
Children with drug-resistant epilepsy	3–18 age Female and male	KD, 12 months	↑ KD retention rate ↑ incidence of adverse reactions	Yang et al. (2022) (168)
Adult with refractory epilepsy	Older than 18 age Female and male	KD, 2 weeks	↓ inflammation ↓ IL-6 and CXCL13 ↑ blood lipids	Dikens et al. (2023) (169)
Children with refractory epilepsy	1–18 age Female and male	KD, 3 months	↓ seizure ↓ CCL 7, CCL 21, CCL 22, CCL 25, CCL 27, IL-2, IL-10, CX3CL1 and MIF	Wickström et al. (2021) (170)

TRF, time-restricted feeding; EEG, electroencephalogram; PTZ, pentylenetetrazol; I/O, input/output; KA, kainic acid; MES, maximal electroshock; maximal dentate activation; PS, population spike; AD, Atkins diet; mAD (MAD), modified Atkins diets; FAST-KD, fasting initiation ketogenic diet; GRAD-KD, gradual initiation; LGIT, low glycemic index therapy; n.s, data has no significance.

### 4.2 Depression and anxiety

Depression, also known as major depressive disorder (MDD), is a psychological disease that involves a persistently depressed mood, lack of energy, anhedonia, sleep disturbances, or even suicidal thoughts. Depression can occur independently at times of significant misfortune, but can also represent a psychological complication of other neurological diseases, especially neurodegenerative diseases, such as AD, PD, and MS. Similarly, anxiety is an emotion-related neuropsychological disorder that can lead to substantial brain damage. As with depression, anxiety can be associated with depressive disorder and other neurological diseases, such as AD, PD, and MS (171, 172). Studies have shown that patients suffering from either of these two diseases could experience emotional eating disorders that usually lead to an excessive body mass index (BMI) (BMI > 23.9) (173–175). Thus, we can speculate that DR could represent a non-pharmacological treatment option.

#### 4.2.1 Preclinical and clinical studies of depression/anxiety

Preclinical studies investigating how DR might influence depression and anxiety are very limited. However, in a study focused on the function of ghrelin and its receptor (growth hormone secretagogue receptor 1a, GHS-R1a), the authors provided evidence that acute but not chronic calorie restriction could provide protection against anxiety-like and despair-like behaviors in a GHS-R1a-dependent mechanism (176). In another study, Li et al. (177) showed that acute fasting exerted antidepressant-like effects by regulating the CREB pathway and 5-HT2 receptors. Interestingly, contradictory findings were reported by Govic et al. (178) in that rats in a long-term 25% calorie restriction group showed an anxiolytic behavioral profile across their whole adulthood. In another study, Sussman et al. investigated how ketogenic diets influenced the susceptibility to depression and anxiety in adult mice. Analysis showed that adult mice receiving a ketogenic diet tended to give birth to offspring exhibiting reduced susceptibility to anxiety and depression (179). Although there is a lack of direct data at present, the link between DR and its anti-inflammatory effects could suggest a possible underlying mechanism linked to the complicated relationships between anxiety, depression, and neuroinflammation (180–184).

Some clinical studies focused on the changes in mood generated by DR in depressive women who were either overweight or obese. Applewhite et al. (185) reviewed clinical research published over the last few decades. Findings from the clinical data of 25 trials corroborated each other and led to a relatively uniform conclusion that a low-calorie diet could ameliorate depressive symptoms from baseline to post-treatment in women who were overweight or obese (185). Another study summarized 14 clinical studies involving 562 individuals and stated that intermittent fasting exerted a positive effect on depression scores but did not modify anxiety (186). Due to the limited body of clinical data, DR is not yet a recommended therapeutic approach for the treatment of depression and anxiety. More preclinical and clinical evidence, including methodological evaluation and assessment of the safety and risk of DR, is urgently required. Table 8 provides a summary of all preclinical and clinical studies referring to depression and anxiety.

**Table 8:** All reviewed preclinical and clinical studies investigating the influence of DR in depression and anxiety.

Animal	DR method	Findings	Reference
Depression and anxiety preclinical studies			
C57BL/6J mice GHS-R1a knockout mice	Chronic CR: 20% CR, 24 days	↓ depressant-like and anxiety-like effects	Lu et al. (2019) (176)
ICR mice	Acute fasting (3 h, 6 h, 9 h,18 h)	↓ depressant-like effects ↑ corticosterone levels	Li et al. (2014) (177)
Wistar rats	25% CR, around 10 months	↑ anxiety-like effects	Govic et al. (2022) (178)

ICR, imprinting control region.

## 5 Safety and risks

Although the positive effects of DR have been proven in a growing number of research studies, the actual efficacy and safety of intermittent and chronic DR should be verified more carefully in preclinical and clinical studies within the near future. The potential occurrence of side effects and risks (e.g., malnutrition, weight loss, and the loss of bone mass) should be taken into consideration due to the distinct influences of DR at different levels, the tolerance variations of patients, as well as the specific condition of patients suffering from different diseases. For instance, it is worth noting that DR might not be a suitable and recommendable therapeutic approach for ALS due to the existing unfavorable evidence dose counterbalancing the positive effects of DR. Studies have shown that 15–55% of ALS patients suffer from malnutrition during the progression of amyosthenia; this malnutrition could be due to the loss of appetite, difficulty in swallowing (oropharyngeal dysphagia), along with weight loss (187). In this case, clinical trials of DR could be difficult and inappropriate. More importantly, nutritional management should gain prominence in the therapeutic process of ALS. It is important to consider that oropharyngeal dysphagia also occurs in other neurodegenerative diseases. Pizzorni et al. (188) recruited 148 patients suffering from HD, PD, and ALS and investigated swallowing function and nutritional assessment. The results of TOMASS (Test of Masticating and Swallowing Solids) and MAS (Mealtime Assessment Scale) assessments were significantly associated with the risk of malnutrition, and as a result, 67.6% of patients were considered at risk of malnutrition (188). This evidence further indicates that DR may not be suitable for a universal adjuvant therapeutic approach for patients who are already suffering from malnutrition. On the other hand, depression and anxiety are always associated with eating disorders, bulimia nervosa, binge-eating disorders, and anorexia nervosa, as classic symptoms. Studies have found that 13% of patients with depression or anxiety met the basic criteria for an eating disorder, and more than one-third of patients engaged in clinically significant disordered eating behaviors (189, 190). Thus, if DR can be considered as one of the adjuvant therapeutic engagements, there should be a more thorough evaluation system to assess the feasibility of this approach. Finally, the selection of a specific DR protocol requires clear guidance, as different DR approaches may not share a biological equivalence (mainly due to their differential abilities to control calories or specific nutrient contents).

Although DR exerts promising beneficial effects in multiple preclinical and clinical studies, the risks (e.g., possibility of malnutrition, heterogeneous outcome) and safety of this approach should be carefully evaluated.

## 6 Limitations

Although this review aimed to incorporate a representative body of evidence from the most widely utilized experimental models and clinical studies, several inherent limitations should be acknowledged. First, this review predominantly synthesized preclinical studies utilizing rodent models, which, although invaluable, may inherently lack the complexity of human neurological disorders. Key differences include species-specific variations in brain physiology, immune system functionality, and the natural progression of disease (e.g., induced rodent models of neurodegeneration cannot mimic human disorders chronically with multifactorial etiologies). Furthermore, only a limited number of studies involving non-human primates have been published, thus increasing the possibility that relevant findings may have been inevitably overlooked. It is also important to note that this review predominantly focused on mammalian systems; thus, highly informative studies in non-mammalian models (e.g., *Caenorhabditis elegans*, Drosophila), which are able to provide fundamental mechanistic insights, were not included. Notably, non-human primates (e.g., the rhesus monkey) have also been used in DR research due to their similarity to humans. However, published studies primarily focused on the effects of DR on general aging (45, 191, 192) rather than its role in the specific neurodegenerative or neurological disorders discussed in this review. Therefore, this aspect of research was not included in our review. It is also critical to recognize that many preclinical models, including non-human primates, do not spontaneously develop the full pathological spectrum equivalent to complex human disorders such as AD or PD. Consequently, it is undeniable that there is a translational gap between these animal findings and the available clinical data. In the clinical realm, on the other hand, human studies remain relatively limited in scope and rigor, often characterized by heterogeneity in study design, patient populations, intervention protocols, potential risks, and safety evaluation, as well as being potentially statistically underpowered. These factors collectively hinder the strength of definitive conclusions. It is gratifying that several clinical trials focusing on neurodegenerative diseases are currently underway to confirm efficacy and safety. Further analysis is necessary to compensate for the lack of standardized and large-scale clinical investigations and bridge this translational gap. Thus, although preclinical evidence appears promising, it is still not possible to propose DR as a clinical recommendation without rigorous human trials.

Second, as a narrative review, our approach inherently carries a risk of selection bias. This bias relates to our decision to structure our analysis around neuroinflammation as the central interpretive point and to select disorders that prominently feature this mechanism. Consequently, our literature search, synthesis, and emphasis were inherently led toward supporting and examining this specific thematic framework. While we aimed to conduct a comprehensive and representative survey of the literature throughout the mainstream databases, our synthesis and emphasis on certain pathways or disease examples may have been influenced by our interpretative framework (our thematic focus on neuroinflammation and classic neurodegenerative diseases). This approach may have potentially underrepresented or omitted other forms of relevant literature, including negative findings or work in less well-cited research areas. Moreover, other critical pathways, including metabolic regulation, autophagy, mitochondrial function, and neuronal excitability, are acknowledged but not systematically introduced in this review due to the thematic choice and the length of this review.

Finally, to maintain a focused scope, this review primarily centered on classic neurodegenerative diseases and other selected neurological conditions. This necessarily excluded discussion of other major categories of neurological disorders where neuroinflammation also plays a pivotal role, such as cerebrovascular diseases (e.g., vascular dementia, stroke), traumatic brain injury (TBI), and neurodevelopmental disorders (e.g., autism spectrum disorder, cerebral palsy). The role of DR in these contexts may share a different fundamental mechanism and needs to be carefully considered in terms of feasibility and translational relevance. Therefore, a separate and in-depth investigation is needed to gain a sufficient illustration of these diseases. Thus, in this review, these diseases were not discussed in detail due to the length and focused scope of this review.

In summary, although this review aimed to synthesize key insights into DR as a neuroprotective strategy, the limitations of our work still highlight the necessity for more standardized preclinical models, large-scale clinical trials, and expanded research into understudied neurological disorders.

## 7 Conclusion and future perspectives

There is a continuous and sustainable growth trend for neurodegenerative diseases, the aging population, and other age-related chronic conditions. Aging-related health issues and obesity are no longer unique to advanced countries; these problems are rapidly spreading to developed countries. This results in costly pandemic health-related problems and chronic disease induced by excessive calorie intake. Chronic geriatric diseases include myocardial infarction, high blood pressure, AD, PD, vascular dementia, and type 2 diabetes. More than 50% of the population of adults over 65 years of age suffer from two or more geriatric diseases (193). For instance, dementia, a typical symptom associated with neurodegenerative diseases, now affects over 55 million patients worldwide. As the aging population increases globally, this number will reach 131 million by 2050 (194). Due to the complicated and refractory nature of neurodegenerative diseases, finding alternative therapeutic approaches is of paramount importance.

As we have summarized in this review, accumulating evidence implies that the pathological brain damage and behavioral changes associated with neurological diseases such as AD, PD, MS, ALS, HD, and epilepsy could be influenced by nutrient-sensing pathways. Thus, targeting dietary structures and habits could represent a promising therapeutic strategy. Specific dietary manipulations have revealed obvious positive effects on pathophysiological mechanistic modulation related to the inflammatory and metabolic changes that have already been identified in the most prevalent neurodegenerative diseases. For instance, restricting the timing or amount of daily food could improve metabolic function in the brain and attenuate the cognitive deficit of patients with AD. On the other hand, reducing the consumption of specific nutrients could also fulfill the regulatory effects of DR due to nutritional modulation of the gut microbiome. The ketogenic diet (a low-carbohydrate and high-fat diet) has been proven to be beneficial to patients (especially pediatric patients) with drug-resistant epilepsy. Based on our current knowledge of DR, several mechanisms have been identified, including nutrient-sensing pathways, neuroinflammation, oxidative stress, the process of autophagy, and insulin sensitivity; these have all been associated with the functional phenotype, including microglial activation, abnormal protein accumulation, synaptic dysfunction, and neuronal death. The possible link between impaired brain metabolism and inflammatory response could be addressed by considering the metabolic changes influenced by DR and other dietary manipulations. From preclinical and clinical perspectives, pathological changes in multiple neurodegenerative and neurological diseases have been proven to be associated with Akt, GSK3β, AMPK, mTOR, and inflammation-related microglia activation in animal models of AD, PD, MS, ALS, epilepsy, and anxiety. Considering the preclinical and clinical evidence together, we cannot assume that all DR modalities are biologically equivalent. Furthermore, current clinical evidence does not yet support DR as a standard therapeutic recommendation across the diseases herein, especially when risks and safety issues still need to be fully investigated.

Animal experiments and human trials have provided encouraging results for DR in terms of its therapeutic effects on neurodegenerative and neurological diseases. The complex interactions between different types of molecular changes caused by DR in the brain–gut axis are being discovered. As the intersection between nutrition, metabolism, and neuroinflammation carries immense hope, the positive effects of DR appear to be clear and solid, although biological equivalence across DR approaches may differ. Though this review aimed to synthesize key insights into DR as a neuroprotective strategy, its limitations still highlight the requirement for more standardized preclinical models, large-scale clinical trials, and expanded research into understudied neurological disorders. Furthermore, future research should prioritize the investigation of biological mechanisms that lead to the heterogeneous outcomes of DR across different neurological disorders. Understanding why DR provides protection for some conditions but fails or even shows a harmful effect in others (e.g., ALS) is crucial if we are to develop personalized and disease-specific DR protocols or nutritional modulation strategies. With the improvement and refinement of DR research, personalized DR regimens, such as fasting-mimicking diets tailored to APOE ε4 carriers with mild cognitive impairment, or customized time-restricted eating paired with anti-inflammatory nutrient supplementation for PD, will overcome the limitations of one-size-fits-all approaches, maximizing neuroprotective efficacy while minimizing risks such as malnutrition in vulnerable elderly populations. Furthermore, to gain a more complete understanding of the metabolic-inflammatory axis in the brain, future work should also systematically compare and contrast models of dietary restriction with those of dietary excess (e.g., a high-fat diet) and metabolic disease (e.g., type II diabetes). Along with the continuous investigation of DR, we expect that comprehensively designed DR approaches will be finally established as a safe and low-cost adjuvant to targeted pharmaceuticals, gene therapies, and neurorehabilitation, thus reshaping the landscape of preventive and therapeutic care for neurodegenerative and other neurological diseases.
